# How resilient is the general population to heatwaves? A knowledge survey from the ENHANCE project in Brussels and Amsterdam

**DOI:** 10.1186/s13104-016-2305-y

**Published:** 2016-11-28

**Authors:** Joris Adriaan Frank van Loenhout, Debarati Guha-Sapir

**Affiliations:** School of Public Health, Institute of Health and Society, Centre for Research on the Epidemiology of Disasters, Université catholique de Louvain, Clos Chapelle-aux-Champs 30, 1200 Woluwé-Saint-Lambert, Brussels, Belgium

**Keywords:** Heat, Heat plan, Risk group, Protective measure, Awareness raising

## Abstract

**Background:**

Studies have shown an increase in mortality and morbidity during heatwaves, especially among the elderly. We assessed the knowledge of the general population of Brussels and Amsterdam on groups at risk and protective measures for heat-related health effects.

**Results:**

Six locations with mixed populations were selected in each city. Passer-by’s in both cities were asked to participate in a short survey. Respondents in Brussels (n = 120) had significantly more knowledge on risk groups and protective measures than respondents in Amsterdam (n = 133). In both cities, individuals with higher education had better knowledge on risk groups and protective measures than individuals with lower education.

**Conclusions:**

Efforts at heat-awareness raising must be strengthened, especially in Amsterdam, and public health actions should effectively target vulnerable groups with lower education in both cities.

**Electronic supplementary material:**

The online version of this article (doi:10.1186/s13104-016-2305-y) contains supplementary material, which is available to authorized users.

## Findings

### Background

Exposure to heatwaves can lead to harmful effects in individuals. Globally, studies have shown an increase in mortality and morbidity among the elderly during heatwaves [1], and an increase in heat-related morbidity among small children [2], although other risk groups are also described (e.g. patients, socially isolated individuals). One of the most severe examples of a heatwave in Europe occurred in 2003, when over 15,000 individuals died in France alone [3]. Heatwaves are also set to increase in the coming years in Western Europe, both in frequency and intensity, due to climate change [4], threatening to aggravate the health situation of the community even further.

Following the 2003 heatwave, several European countries developed heatwave early warning systems or national heat plans [5]. These plans aim at reducing the avoidable human health consequences of heatwaves. Although the main purpose is to establish the role of professionals (e.g. in elderly care facilities, or general practitioners) during a heatwave, national heat plans also contribute in increasing awareness of heat risks in vulnerable groups and their care providers. The messages are channelled through community professionals and indirectly through the media.

Both Belgium and the Netherlands have a heat plan in place since 2005 and 2007, respectively [6, 7]. We assessed the knowledge of the general population of these countries on the groups at risk and protective measures for heat-related health effects, which provides an indication of the resilience of the population on this topic. Both heat plans address these issues, which should be generally known by the community, as most individuals either belong to a risk group or have one in their direct environment. Crucially as well, effective public health actions against heat are highly dependent on the popular perceptions that make up the health strategy, such as attitudes to heat, known protective measures and knowledge of risk groups. We hypothesised that there was no difference in knowledge level between the two cities.

### Methods

This was a cross-sectional observational study, for which a two-page questionnaire was designed (Additional files 1, 2). It included questions on demographics, the respondents’ familiarity with the heat plan, risk groups for heat (open question) and protective measures against adverse heat-related health effects (open question). In addition, respondents were asked whether they considered themselves sensitive to extreme heat and whether the government was doing enough to raise awareness on this issue.

We selected Brussels and Amsterdam for our study, since they are the capitals and biggest cities of Belgium and the Netherlands, respectively, and the effects of heatwaves are stronger in urban areas due to the urban heat island effect. The study was conducted on 19–20 August 2015 within Brussels, and on 23–25 September 2015 within Amsterdam. Six locations with mixed populations in terms of socio-economic background were selected in each city. Different socio-economic indicators were available for both cities, namely average income per capita for Brussels and status score for Amsterdam (Table 1). Even though these indicators are not comparable between the two cities, they provide an indication of the variation in socio-economic diversity within each city. Passer-by’s were asked to participate, and provided verbal consent. Interviews were conducted in Dutch, French and English. Individuals older than twelve years, living in Brussels or Amsterdam and speaking one of the survey languages were eligible for inclusion in the study. Tourists and workers not living in the city were excluded. Approximately 20 interviews were carried out per location.

**Table 1:** Description and indication of socio-economic status of locations per city where the survey was conducted

Brussels			
Municipality^a^	Survey location type	Average income per capita^b^	Ranking^d^
Ixelles	Shopping street	€15,068	2nd quartile
Etterbeek	Park	€13,746	3th quartile
Etterbeek	Tram/bus connection	€13,746	3th quartile
Brussels	Shopping street	€12,079	3th quartile
Anderlecht	Outdoor market	€11,356	4th quartile
Saint-Gilles	Tram/bus connection	€11,718	4th quartile

Amsterdam			
Area^a^	Survey location type	Status score^c^	Ranking^d^
Museumkwartier	Park	2.62	1st quartile
Landlust	Shopping centre	−0.66	4th quartile
Apollobuurt	Shopping street	2.88	1st quartile
Oude Pijp	Outdoor market	0.87	2nd quartile
Bijlmer Oost	Shopping street	−2.2	4th quartile
IJburg	Shopping street	1.59	1st quartile

^a^The Brussels Capital Region consists of 19 municipalities, while Amsterdam consists of 71 areas, all of them defined by unique zip codes

^b^The average income per capita over the year 2013, as determined by Statistics Belgium [12]

^c^The status score of each area, indicating the social status in comparison to other areas. This figure is a compilation of educational level, income and work situation of the inhabitants, and was determined by the Social and Cultural Planning Agency of the Netherlands over the year 2014 [13]

^d^Ranking for each municipality in Brussels is in comparison to all 19 municipalities in the Brussels Capital Region. Ranking for each area in Amsterdam is in comparison to all 3541 areas in the Netherlands

Results from the questionnaire were compared between Brussels and Amsterdam, using Pearson Chi square tests and Independent Samples *t* tests. A sensitivity analysis was conducted on the combined data using logistic regression, to assess whether educational level was associated with knowledge on risk groups and protective measures. This was done only for groups and measures where the familiarity was at least 10% (in one city or both). Another sensitivity analysis was carried out, to compare the proportion of elderly (≥65 years of age) who named the elderly as a risk group to the proportion who considered themselves sensitive to heat. A p value of <0.05 was considered to be statistically significant, based on two-sided tests. Data were analysed using the software SPSS for Windows (version 22).

### Results

#### Survey outcomes

The samples were 120 and 133 for Brussels and Amsterdam, respectively, and there were no significant differences in gender, age and educational level (Table 2).

**Table 2:** Characteristics of respondents, their knowledge on risk groups and protective measures for heat-related health effects and their opinion on government activities

	Brussels	Amsterdam	Difference^a^
	N = 120	N = 133	p value
*Demographics of respondents*			
Male gender %	46.7	49.6	0.642
Average age in years (sd)	45.5 (18.1)	43.5 (19.6)	0.399
Education %^b^			0.515
Lower	20.0	19.1	
Medium	19.2	25.2	
Higher	60.8	55.7	
*Knowledge of respondents*			
Familiarity with existence of the heat plan %			0.314
Yes	39.2	33.1	
No	60.8	66.9	
Familiarity with risk groups for heat %^c^			
Elderly	87.5	69.2	*<0.001*
Young children/babies	64.2	43.6	*0.001*
Sick individuals/patients	35.0	36.1	0.856
Socially isolated individuals	9.2	0.0	*<0.001*
Pregnant women	4.2	3.8	0.868
Obese individuals	2.5	6.8	0.111
Individuals who perform a lot of physical effort	0.8	1.5	0.623
Don’t know/only non-formal risk group	10.0	24.8	*0.002*
Familiarity with protective measures for heat %^c^			
Drink fluids	80.8	59.4	*<0.001*
Avoid heat/sun^d^	58.3	64.7	0.301
Adjust clothing^e^	28.3	36.1	0.188
Cool the body^f^	22.5	18.8	0.467
Use fan or airconditioning	18.3	24.8	0.212
Keep windows closed	16.7	6.8	*0.014*
Avoid physical activity	11.7	15.0	0.432
Adjust diet	9.2	8.3	0.801
Visit green areas	8.3	1.5	*0.011*
Use sunscreen	6.7	25.6	*<0.001*
Don’t know	1.7	0.0	
*Opinion of respondents*			
Sensitive to heat %			*<0.001*
Very much	31.7	12.0	
Somewhat	13.3	27.8	
Not at all	54.2	59.4	
Don’t know	0.8	0.8	
Sufficient awareness by government %			*<0.001*
Too little	46.7	28.6	
Just enough	38.3	31.6	
Too much	2.5	3.8	
Don’t know	12.5	36.1	

^a^Differences between groups were tested using an Independent Samples *t* test for age, and Pearson Chi square tests in all other cases. Significant differences are in italics

^b^Educational level was categorised out of five groups in Brussels: (1) none, (2) primary school, (3) secondary school technical/professional (lower education), (4) secondary school general (medium education), (5) college/university (higher education); and seven groups in Amsterdam: (1) none, (2) primary school, (3) lower vocational education, (4) general secondary education (lower education), (5) secondary vocational education, (6) senior general or pre-university education (medium education), (7) college/university (higher education)

^c^Familiarity with risk groups and protective measures for heat-related health effects were asked as open questions, where respondents could provide as many answers as they wanted. Answers were later grouped in the above-mentioned categories

^d^Avoid heat/sun includes the answers ‘stay inside’ and ‘stay in the shade’

^e^Adjust clothing includes the answer ‘wear a hat’

^f^Cool the body includes the answers ‘swim’ and ‘take a shower’

Knowledge on the heat plan did not significantly differ between the cities (Table 2), although 57% of the respondents in Brussels familiar with the heat plan knew that it was last activated in 2015, compared to 28% in Amsterdam. Respondents in Brussels had significantly more knowledge on the elderly, children and socially isolated individuals as risk groups. Respondents in Amsterdam were more often not able to name any risk group, or named a group not formally considered as such (most often ‘individuals with light skin’). Respondents in Brussels also had significantly more knowledge on drinking fluids, keeping the windows closed and visiting green areas as protective measures. Respondents in Amsterdam more often proposed ‘using sunscreen’ as an answer.

Respondents in Brussels considered themselves more sensitive for heat, and more often had the opinion that the government does not raise enough awareness on this topic (Table 2). Respondents in the Netherlands more often replied ‘don’t know’ to the last question, mainly because they were simply not aware of any activities that the government takes with respect to heat.

#### Sensitivity analyses

Individuals with lower education had less knowledge of the elderly and children as risk groups, or named groups not formally considered at risk (Table 3). Individuals with higher education were significantly more aware of drinking fluids as a protective measure compared to those with medium or lower education. Also, individuals with higher education recognised more frequently the importance of avoiding heat/sun compared to those with lower education.

**Table 3:** The relationship between educational level of respondents and knowledge on risk groups and protective measures for heat-related health effects

	Medium education	Lower education
	OR (95% CI)^a^	OR (95% CI)^a^
Lower familiarity with risk groups for heat		
Elderly	2.0 (0.9–4.1)	*3.1 (1.5–6.5)*
Young children/babies	1.9 (1.0–3.5)	*6.4 (3.0–13.7)*
Sick individuals/patients	0.9 (0.5–1.7)	1.5 (0.7–3.0)
Don’t know/only non-formal risk group	0.7 (0.3–1.7)	*0.4 (0.2–0.8)*
Lower familiarity with coping measures for heat		
Drink fluids	*2.2 (1.1–4.3)*	*2.0 (1.0–4.0)*
Avoid heat/sun	0.9 (0.5–1.8)	*2.4 (1.2–4.6)*
Adjust clothing	1.3 (0.7–2.5)	2.0 (0.9–4.2)
Cool the body	1.2 (0.6–2.7)	0.9 (0.4–2.0)
Use fan or airconditioning	2.1 (0.9–5.1)	0.8 (0.4–1.6)
Keep windows closed	2.1 (0.7–6.3)	1.4 (0.5–3.9)
Avoid physical activity	1.6 (0.6–4.1)	1.6 (0.6–4.6)
Use sunscreen	0.5 (0.2–1.1)	0.8 (0.3–2.0)

*OR* odds ratio, *CI* confidence interval

^a^Differences between educational levels were tested using logistic regression, where ‘higher education’ was the reference group. Statistically significant ORs are in italics

The proportion of elderly (≥65 years of age) who named the elderly as a risk group for heat was 86.4%. The proportion of elderly who considered themselves somewhat or very sensitive to heat was 63.6%.

### Discussion

Our study suggests that respondents in Brussels had greater knowledge on their national heat plan, and risk groups and protective measures for heat-related health effects than respondents in Amsterdam. Results from Amsterdam indicated some confusion between the terms ‘exposure to heat’ and ‘exposure to sunlight’. Although the climates of the two cities are very similar, respondents in Brussels considered themselves more sensitive to heat and felt that the government should be more proactive on public education.

Although heat-protection messages are fairly straightforward and are usually covered by the media before a heatwave happens [8], our results suggest that they do not effectively reach individuals with lower education. Current public health activities to raise awareness on heat should further strengthen their efforts to reach high-risk groups with lower education in both these countries.

Out of the elderly respondents, more than 20% were aware that the elderly are a risk group for heat, but did not consider themselves sensitive to heat. This implies that, even when the knowledge level of individuals is good, there can be a misperception of people’s own risk.

Two other studies are relevant in the context of our study, both from the UK. First, a qualitative study among elderly persons indicated that most were able to provide appropriate examples of behaviours to reduce the effects of heat [9]. Second, a study concluded that education was positively correlated to heat-prevention action [10], confirming the findings of our study.

Our results are not necessarily representative for the cities of Brussels and Amsterdam, but they give a first indication of the knowledge and perception in these cities. There was a difference in weather circumstances between the two surveys (warm and sunny in Brussels, versus wet in Amsterdam) and in timing (the surveys were carried out 5 weeks apart), which might have had an impact on the perception of individuals on heat, although we do not expect this to influence the individuals’ knowledge level.

### Conclusions

Our study results suggest that efforts at heat-awareness raising must be strengthened, especially in Amsterdam and perhaps in other cities of the Netherlands. We encourage a dialogue between representatives for the heat plans in Belgium and the Netherlands, to exchange their strategies on awareness raising with respect to heat in the population. Secondly, for both Brussels and Amsterdam, public health actions should effectively target vulnerable groups with lower education. This could possibly be better achieved by active collaboration between public health authorities and media that are popular among individuals with lower education, such as certain websites or news broadcasts. Lessons on how to involve communities can also be learnt from the Climate Vulnerability and Capacity Analysis Handbook, such as Participatory Learning for Actions (PLA) tools [11].

## Additional files

**Additional file 1.** English version of questionnaire used in Brussels.

**Additional file 2.** English version of questionnaire used in Amsterdam.
