# Supplementary material for: How resilient is the general population to heatwaves? A knowledge survey from the ENHANCE project in Brussels and Amsterdam
Source: BMC Res Notes. 2016 Nov 28;9:499. doi: 10.1186/s13104-016-2305-y (PMC5127085; doi:10.1186/s13104-016-2305-y)
Supplement: Supplementary file 2 — Additional file 2. English version of questionnaire used in Amsterdam. [file 13104_2016_2305_MOESM2_ESM.docx]

**Annex 2: English version of questionnaire used in Amsterdam**

**Introduction**

Location: Date:

Interviewer : ID:

Good morning / afternoon. We are researchers from the Université Catholique de Louvain, and we are trying to improve services with respect to extreme heat in Amsterdam. May I ask you some questions about this? This will take around 5 minutes.

Thank you for your time. This study is on the perception of the general public on extreme heat and the “National Heat Plan”, to find out to what extent people are familiar with it. This is important, also since there will be an increase in the number of heatwaves due to climate change. This study will be carried out in both the Netherlands and Belgium, as part of a European project.

**Questions**

1. Do you live in Amsterdam?

Yes

No 🡪 **If no, not eligible for this study!**

1. For how many years have you lived in Amsterdam?
2. Are you aware of the existence of a “National Heat Plan” in the Netherlands, which is activated if high temperatures are predicted for several days?

Yes 🡪 go to question 4

No, but I know that some measures exist 🡪 go to question 4

No 🡪 go to question 6

1. Where did you hear about this plan?

Television

Radio

Newspaper

Internet / social media

Through relatives / friends

 In the street

Other way, namely

1. Do you know in which year the heat plan was activated the last time?

1. Some people are more sensitive to extreme heat. Can you name some groups of people who have a higher risk for health effects due to extreme heat? (**Don’t provide respondent with the options below! But please select the option(s) that best approximate the responder’s answer**)

Elderly

Very young children / babies

People who take certain kind of medication / patients (sick people)

People who are socially isolated (homeless persons / migrants)

People who perform a lot of physical efforts (sports / construction work)

 Other, namely

1. There are ways in which you can protect yourself from extreme heat. Do you know some things you could do to prevent health effects from extreme heat? (**Don’t provide respondent with the options below! But please select the option(s) that best approximate the responder’s answer**)

Increase consumption of fluids (water / soft drinks)

Stay inside during the warmest times of the day

Keep windows closed when the temperature outdoors is higher than indoors

Adjust your clothing (light materials, light colours)

Visiting green areas (forest, park, etc.)

Cooling your body, e.g. by taking a shower, bath or swimming

Using a fan or airconditioning

Avoid physical activity (sports)

 Other, namely

1. Do you consider yourself sensitive for extreme heat?

Very much  Somewhat  Not at all

1. Do you think the government raises enough awareness for extreme heat?

Too little  Just enough  Too much

1. Do you have any other remarks / comments on extreme heat that might be of interest for us?
2. Gender

Male  Female

1. What is your age?
2. What is your educational level?

None

Completed primary school

Completed lower vocational education

Completed general secundary education

Completed secondary vocational education

Completed senior general or pre-university education

Completed college or university studies

1. What is your nationality?

**Closure**

Thank you very much for your participation. If you want to receive a short summary of our results, you can provide us with your email address. This will not be used for any other purpose.

Interviewer comments
